# Intelligent Classification Method for Rail Defects in Magnetic Flux Leakage Testing Based on Feature Selection and Parameter Optimization

**DOI:** 10.3390/s25133962

**Published:** 2025-06-26

**Authors:** Kailun Ji, Ping Wang, Yinliang Jia

**Affiliations:** College of Automation Engineering, Nanjing University of Aeronautics and Astronautics, Nanjing 210016, China; zeitping@nuaa.edu.cn (P.W.); jyl@nuaa.edu.cn (Y.J.)

**Keywords:** rail defect detection, magnetic flux leakage (MFL) testing, feature selection, intelligent classification, model optimization, imbalanced data

## Abstract

This study addresses the critical challenge of insufficient classification accuracy for different defect signals in rail magnetic flux leakage (MFL) detection by proposing an enhanced intelligent classification framework based on particle swarm optimized radial basis function neural network (PSO-RBF). Three key innovations drive this research: (1) A dynamic PSO algorithm incorporating adaptive learning factors and nonlinear inertia weight for precise RBF parameter optimization; (2) A hierarchical feature processing strategy combining mutual information selection with correlation-based dimensionality reduction; (3) Adaptive model architecture adjustment for small-sample scenarios. Experimental validation shows breakthrough performance: 87.5% accuracy on artificial defects (17.5% absolute improvement over conventional RBF), with macro-F1 = 0.817 and MCC = 0.733. For real-world limited samples (100 sets), adaptive optimization achieved 80% accuracy while boosting minority class (“spalling”) F1-score by 0.25 with 50% false alarm reduction. The optimized PSO-RBF demonstrates superior capability in extracting MFL signal patterns, particularly for discriminating abrasions, spalling, indentations, and shelling defects, setting a new benchmark for industrial rail inspection.

## 1. Introduction

As critical infrastructure for railway transportation, rails directly determine the safety and efficiency of railway systems. During long-term operation, the rail surface is prone to various types of defects—including abrasions, spalling, indentations, and shelling—due to rolling contact fatigue (RCF) with vehicle wheelsets [1]. These defects compromise the rail’s wear resistance and fatigue strength while significantly altering its electromagnetic properties, ultimately threatening both service life and operational safety.

Conventional ultrasonic testing methods face inherent limitations in their detection principles, making it challenging to achieve efficient and accurate inspection of rail surface defects [2]. Consequently, substantial manual field inspections remain necessary in actual railway operations, failing to meet the demands of modern railway development.

To address this issue, the China Academy of Railway Sciences, in collaboration with Professor Wang Ping’s team from Nanjing University of Aeronautics and Astronautics and Gemac Engineering Machinery Co., Ltd., jointly developed the GTC-80II large-scale rail inspection vehicle. Equipped with an advanced electromagnetic detection system for rail head defects, this vehicle can effectively identify abrasions, spalling, indentations, shelling, and other defects on the rail running surface. Based on high-speed DC leakage flux testing technology, the system achieves inspection speeds up to 350 km/h under laboratory conditions. Compared with traditional ultrasonic techniques, it demonstrates superior advantages including higher speed, improved accuracy, elimination of couplant requirements, and the capability to detect near-surface defects (within 8 mm depth).

Nevertheless, despite the outstanding performance of this electromagnetic detection system in defect identification, significant challenges persist in the classification of electromagnetic signals. Currently, there lacks an effective methodology to accurately recognize and differentiate various types of defect signals.

In MFL testing specifically, rail defect classification faces three unique challenges [1]:

**Signal Complexity:** The superposition of leakage fields from adjacent defects (e.g., clustered spalling) creates nonlinear signal interference, with studies showing up to 40% amplitude modulation when defect spacing < 10 mm.

**Feature Ambiguity:** Critical MFL features (peak spacing, skewness) exhibit <15% separability between abrasions and shelling defects due to similar depth-width ratios.

**Environmental Noise:** Field measurements confirm vibration-induced baseline drift can obscure >30% of defect signals’ characteristic peaks. These factors collectively limit conventional MFL classification accuracy to <70% in operational conditions.

**Figure 1:**
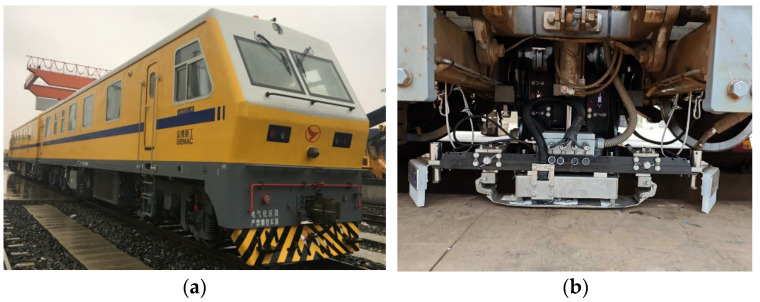
Rail magnetic flux leakage detection system: (**a**) GTC-80 rail inspection vehicle. (**b**) Rail magnetic flux leakage (MFL) detection probe.

This study, therefore, aims to develop a deep learning-based classification model to enhance the accuracy of rail surface defect signal classification, thereby providing more reliable decision support for railway maintenance. See Figure 1.

With the rapid development of modern railway systems, rail defect detection technologies face increasingly stringent challenges. Although existing methods have achieved notable improvements in stability and efficiency, traditional manual classification approaches exhibit evident limitations when processing massive volumes of suspected defect signals. Such experience-dependent subjective judgment not only suffers from inconsistent accuracy and low efficiency but also proves difficult to standardize due to its inherent nonlinearity and strong coupling characteristics [3]. Consequently, developing intelligent automated defect recognition and classification systems holds substantial engineering value for achieving precise rail defect prevention and scientific maintenance, ultimately ensuring railway operational safety.

In practical applications, defect signals collected by leakage flux inspection systems are often contaminated by multiple interference factors. Research indicates that three-dimensional mechanical vibrations during train operation cause signal baseline drift, while electromagnetic interference along tracks introduces high-frequency noise, collectively reducing the signal-to-noise ratio (SNR) of valid signals below 15 dB [4]. Furthermore, rail surface defects typically exhibit the following characteristics: small geometric dimensions, irregular morphologies (e.g., discontinuous distribution patterns of shelling), and significant feature overlap between defect categories (e.g., <10% amplitude difference in magnetic signals between abrasions and spalling). These factors persistently limit the accuracy of conventional threshold-based classification methods.

## 2. Related Works

From a pattern recognition perspective, rail defect classification fundamentally constitutes a nonlinear mapping problem from high-dimensional feature space to discrete category space [5]. Artificial neural networks (ANNs) demonstrate unique advantages in this domain due to their exceptional nonlinear system modeling capabilities. Among them, BP neural networks have become the preferred classifier architecture in engineering applications, owing to their excellent nonlinear approximation properties (theoretically capable of approximating any continuous function with arbitrary precision), modular hardware implementation characteristics, and generalization ability achieved through regularization techniques. Probabilistic neural networks (PNNs) employ Parzen window functions for probability density estimation, requiring only single-pass sample training with O(*n*) time complexity, making them particularly suitable for real-time online classification tasks [6].

However, existing neural network approaches still face unresolved technical challenges: BP networks are prone to local minima and exhibit sensitivity to hyperparameters like learning rates [7]; although PNNs train rapidly, their classification performance heavily depends on smoothing factor selection, with memory consumption growing linearly with training sample size. Moreover, in recent years, biologically inspired spiking neural networks (SNNs) have shown significant potential in machine fault detection tasks. Relevant studies have begun to compare the performance of reservoir artificial neural networks with SNNs, providing a new perspective and methodology for our research [8,9]. These limitations drive ongoing exploration of more advanced intelligent algorithms to address key technical bottlenecks in rail defect classification.

Recent years have witnessed significant advancements in rail defect classification research driven by machine learning and intelligent algorithms. As demonstrated in the literature [10], a Fast R-CNN-based approach was proposed for rail crack detection, where feature extraction and classification of rail defect images achieved high-precision damage identification, confirming the applicability of deep learning in rail flaw detection. Literature [11] employed the Random Forest (RF) algorithm for multi-class prediction of fault data, with results indicating that this method maintains strong robustness even in noisy environments, though it exhibits notable dependence on feature engineering.

With the introduction of optimization algorithms, the literature [12] utilized the Genetic Algorithm (GA) to optimize the kernel function parameters of Support Vector Machine (SVM), thereby improving the accuracy of defect classification, albeit at the cost of increased computational complexity. Literature [13] proposed an RBF neural network model based on Adaptive Particle Swarm Optimization (APSO), which demonstrated fast convergence speed and high classification accuracy on rail defect datasets, providing novel insights into the application of intelligent optimization algorithms for damage prediction. These studies collectively highlight both the promising potential and existing challenges of intelligent algorithms in advancing rail defect detection technologies.

Nevertheless, existing methods still exhibit limitations: traditional BP networks easily converge to local optima with low training efficiency; SVMs incur substantial computational overhead for large datasets; while RBF neural networks possess strong nonlinear fitting capabilities, their center selection and width parameter optimization still rely on empirical adjustment [14]. To address these issues, this study proposes an improved MI-PSO-RBF model, incorporating multimodal information (MI) fusion to enhance feature representation and adaptive PSO-optimized RBF network parameters [15], thereby improving both accuracy and generalization performance in rail defect classification.

## 3. Rail Magnetic Flux Leakage Detection Technology

As a critical non-destructive testing (NDT) method, MFL technology is widely employed for defect detection in ferromagnetic materials [16]. The principle relies on magnetic field induction and material magnetization, where an external magnetic field is applied to the test specimen. Surface or near-surface defects (e.g., cracks, fatigue, or deformations) disrupt the magnetic flux distribution, generating detectable leakage fields. Through advanced signal processing, these anomalies are analyzed to determine defect characteristics (location, geometry, and dimensions). This technology significantly enhances industrial equipment safety by enabling early failure prevention, thereby reducing maintenance costs and production downtime.

### 3.1. Rail Defect Detection Mechanism

**Figure 2:**
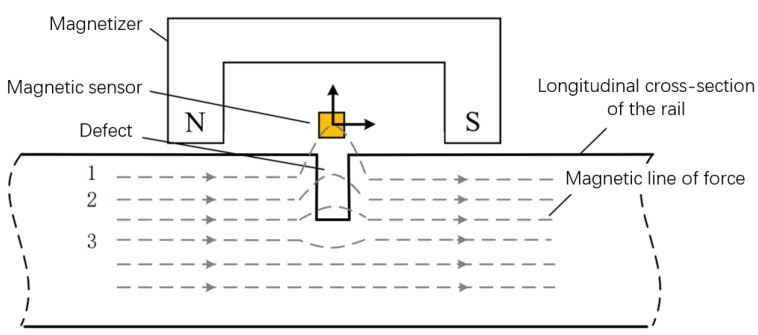
Schematic Diagram of Magnetic Flux Leakage (MFL) Detection Principle.

During rail inspection, localized magnetization ensures magnetic flux lines intersect potential defects. When surface/subsurface defects exist, the permeability contrast between steel and air causes flux leakage at defect sites according to the principle of magnetic flux continuity. The key challenge lies in interpreting these leakage signals for defects with complex morphologies. See Figure 2.

In actual railway lines, rail defects often exhibit irregular shapes with significant dimensional variations, undoubtedly increasing the difficulty of defect classification and quantitative analysis. Nevertheless, different types of defects still demonstrate certain correlations in their effects on magnetic flux leakage (MFL) signals. For instance, defect depth primarily influences signal amplitude, while defect width affects peak spacing and peak area in MFL signals. Meanwhile, defect irregularity is reflected in signal skewness, kurtosis, and other higher-order statistical features. When multiple influencing factors collectively affect defect signals, the resulting complexity makes it impossible to accurately characterize them using simple formulas or analytical functions. To address this challenge, this study adopts a radial basis function (RBF) neural network approach to decouple and analyze defect signals, systematically investigating the relationship between signal characteristics and different defect types to achieve effective classification of MFL signals.

During railway operation, rails serve as critical infrastructure components that endure long-term wheel–rail contact stresses, frictional forces, and various external factors, inevitably leading to the formation of diverse natural defects. These defects not only significantly reduce rail service life but may also pose serious threats to operational safety. Common rail surface defects include rail abrasions, spalling, surface indentations, and gauge corner shelling [17].

### 3.2. Rail Defect Types and Signal Characteristics

In operational railways, four predominant defect types are observed:

1. Rail abrasion (Depth: 0.5–2 mm; Length: 20–100 mm): caused by wheel slippage during acceleration/braking, typically showing 4–20 abrasion marks per rail.
2. Rail spalling (Width: 3–8 mm; Max depth: 3 mm): occurs at curve outer edges, characterized by discontinuous material loss due to rolling contact fatigue.
3. Rail indentation: results from foreign object intrusion, forming isolated or periodic pits on the railhead.
4. Shelling: fatigue-initiated microcracks that propagate into fish-scale patterns, primarily in high-stress zones.

**Figure 3:**
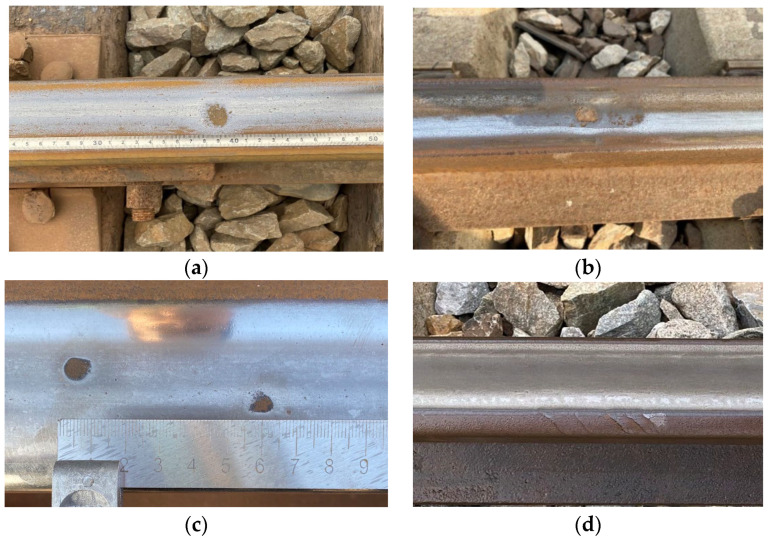
Four typical types of natural rail defects: (**a**) Rail abrasion; (**b**) Rail spalling; (**c**) Rail indentation; (**d**) Shelling.

Given the considerable challenges associated with obtaining diversified defect samples from actual railway tracks, this study proposes a field-verified defect sample collection methodology. Specifically, through comprehensive analysis of acquired suspected defect signals combined with mileage information and marker locations, precise field positioning is achieved for on-site defect verification. While this process enables the acquisition of authentic defect data to a certain extent, the variety and quantity of samples remain constrained by field conditions, proving inadequate to meet the substantial demand for diverse samples required for model training and testing. See Figure 3.

**Figure 4:**
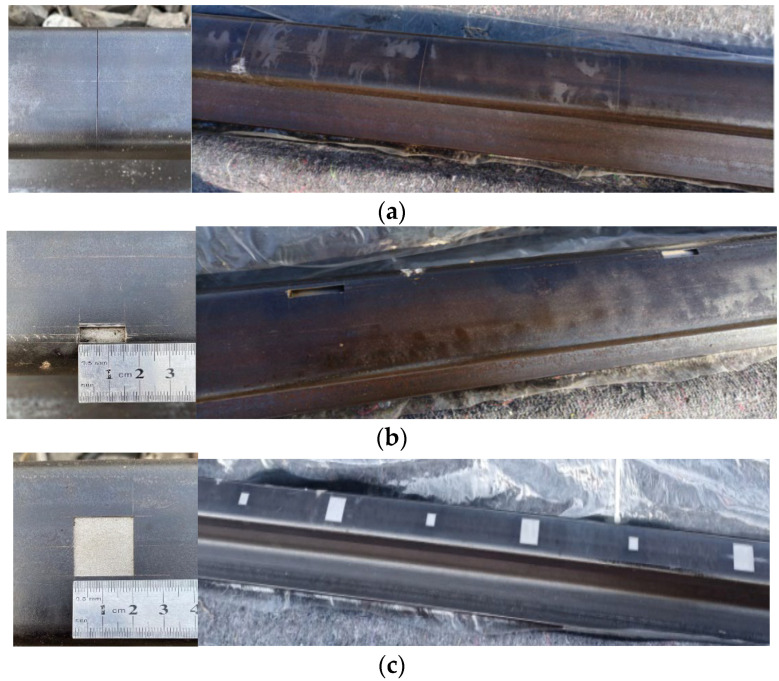
Physical images of four types of artificial defects: (**a**) Physical image of artificial transverse crack defect on rail surface; (**b**) Physical image of artificial spalling defect at gauge corner; (**c**) Physical images of artificial defects—abrasion and indentation on rail surface.

To overcome this limitation and ensure the reliability of the RBF neural network model in defect classification tasks, this study meticulously designed and fabricated a series of artificial defect rail samples. Through extensive experimentation, systematic acquisition of sample data under varying defect types and severity levels was accomplished, establishing a robust data foundation for model training and validation. These artificial defect samples encompass multiple prevalent defect types including cracks, abrasions, spalling, and indentations, with careful dimensional parameter planning and adjustment to comprehensively simulate the complex diversity of defects encountered in operational track conditions. Physical demonstrations of the artificial defect samples are presented as follows. See Figure 4.

## 4. Methodology

### 4.1. Analysis of Magnetic Flux Leakage (MFL) Defect Signal Characteristics

Magnetic flux leakage (MFL) detection technology is widely used in defect detection due to its reliability and well-established principles. By analyzing changes in MFL signals, different types of defects can be effectively identified. Through quantitative analysis of signal characteristics, distinct patterns of various defects can be revealed, which is essential for building an accurate classification system.

Based on existing research and our study on rail surface defects [18], we selected nine key features that effectively distinguish different defect signals:

1. Peak-to-peak value: the amplitude range calculated as the difference between the maximum and minimum signal values.
2. Peak spacing: the interval between two adjacent peaks, commonly used to analyze periodic characteristics of signals.
3. Peak area: the area under a signal peak, which can be calculated by integrating the peak region.
4. Peak-to-peak slope: the slope variation during the transition from one peak’s decline to another peak’s rise, reflecting the signal’s rate of change.
5. Mean value: the arithmetic average of all sample values, representing the central tendency of the signal.
6. Crest factor: the ratio of the signal’s peak value to its RMS value, indicating the relationship between peak amplitude and average power.
7. Kurtosis: a measure of the “tailedness” of a real-valued random variable’s probability distribution, describing the sharpness and heaviness of the signal’s tails.
8. Root Mean Square (RMS): the effective value of a signal, representing its power-equivalent magnitude.
9. Skewness: a measure of the asymmetry of a real-valued random variable’s probability distribution, describing the signal’s distributional bias.

### 4.2. RBF Neural Network Architecture

The Radial Basis Function (RBF) neural network represents an efficient feedforward neural network architecture, whose core principle lies in utilizing radial basis functions as the “basis” for hidden layer units. This unique structure enables nonlinear mapping between the input and hidden layers while maintaining linear mapping between the hidden and output layers. Such distinctive network configuration endows RBF networks with significant advantages in handling nonlinear problems, demonstrating superior convergence speed and nonlinear approximation capabilities compared to the widely used BP neural networks.

The RBF network comprises three fundamental components: the input layer, hidden layer, and output layer. The input layer serves to receive external input signals and transmit them into the network. Assuming the input layer contains m nodes, each node corresponds to one dimension of the input signal. The hidden layer, as the critical component of the RBF network, performs nonlinear transformation of input signals. The number of neurons in the hidden layer typically relates to the complexity of training data. Assuming the hidden layer contains *n* nodes, each node corresponds to a radial basis function. These basis functions, centered on the input signals, generate nonlinear features by computing the distance between input signals and center points. The hidden layer connects to the output layer through weight vector w, and the output layer produces the final network output through linear combination of hidden layer outputs.

In practical applications, the Gaussian basis function—positive definite in arbitrary space—is commonly selected as the hidden layer function for RBF neural networks, as expressed in Equation (1):

(1) $$p_{j}=\exp\left(\frac{{\left\|\left(x-c_{j}\right)\right\|}^{2}}{2\delta_{j}^{2}}\right)\;j=1,2,\ldots ,n$$

where

*p_j_* represents the vector of the *j*-th neuron in the hidden layer;

*x* denotes the neural network input sample;

*c_j_* indicates the center vector of the *j*-th hidden layer node, with dimensionality identical to the input sample;

*δ_j_* corresponds to the width of the *j*-th hidden layer node.

The linear relationship in the output layer of the RBF network is expressed by Equation (2):

(2) $$y=\sum_{j=1}^{n}w_{j}p_{j}$$

where

*y* represents the computed output value of the neural network;

*w_j_* signifies the weight vector between the *j*-th hidden layer neuron and the output layer;

*n* denotes the number of hidden layer neuron nodes.

Although RBF neural networks demonstrate excellent capability in handling nonlinear problems, the determination of network parameters critically influences model outputs. Therefore, the identification of several key parameters—including the RBF center *c_j_*, normalization constant, and weight coefficients *w_ij_* between the hidden and output layers—becomes particularly crucial.

### 4.3. Feature Selection Using Mutual Information

In magnetic flux leakage (MFL) detection technology, the extracted signal features typically exhibit multi-dimensional characteristics. For instance, nine distinct feature values derived from MFL signals constitute continuous feature sets for each sample, including but not limited to peak-to-peak amplitude, peak spacing, peak area, and other relevant parameters. However, directly employing all these continuous features as inputs for artificial neural networks not only consumes substantial computational resources but may also compromise prediction accuracy due to interference from irrelevant features.

To address this challenge, we employ the Mutual Information (MI) criterion for feature selection and dimensionality reduction [19]. Mutual information serves as a robust metric for quantifying the mutual dependence between two variables, particularly suitable for evaluating the correlation between continuous features and discrete defect categories. The calculation formula for mutual information is expressed as follows:

(3) $$I\left(X;Y\right)=\sum_{y\in Y}\sum_{x\in X}P\left(x,y\right)\log_{2}\left(\frac{p\left(x,y\right)}{p\left(x\right)p\left(y\right)}\right)$$

where

- *p*(*x*, *y*) represents the joint probability of feature *X* taking value *x* and defect category *Y* taking value *y*;
- *p*(*x*) denotes the marginal probability of feature *X* taking value *x*;
- *p*(*y*) indicates the marginal probability of defect category *Y* taking value *y*.

In practical applications, these probabilities can be estimated by analyzing the frequency distribution within the dataset. For continuous features, probability density functions are typically estimated using kernel density estimation methods, from which both marginal and joint probabilities can be derived.

By computing mutual information values between defect signal features and defect categories, we can identify the most relevant features for defect classification, which are subsequently utilized for predictive model training and validation. This approach significantly enhances prediction accuracy while reducing computational overhead.

The mutual information calculation can alternatively be implemented through a parameter-free *k*-nearest neighbor (KNN) based method. This technique employs the maximum Euclidean distance in both *X* and *Y* directions as the criterion for nearest neighbor selection, followed by statistical counting and probability density estimation. This methodology not only effectively handles the complex relationships between continuous features and discrete labels but also preserves the most critical information during feature selection, thereby improving both model performance and computational efficiency.

To address potential limitations of Mutual Information (MI) in handling nonlinear or redundant features due to its reliance on probability density estimation, this study additionally compares the feature screening performance of ReliefF and Recursive Feature Elimination (RFE). ReliefF evaluates feature discriminability based on nearest-neighbor samples, making it more suitable for capturing nonlinear relationships, while RFE iteratively removes redundant features by optimizing classifier performance, demonstrating stronger robustness for high-dimensional data.

Through these methods, we can efficiently extract the most valuable features from high-dimensional data, establishing a robust foundation for subsequent neural network training and defect classification tasks.

### 4.4. PSO Algorithm for Parameter Optimization

The particle swarm optimization (PSO) algorithm simulates the foraging behavior of bird flocks [20], where each particle’s position represents a potential solution. During iterations, particles move toward their individual historical best positions. In an M-dimensional search space, *x_i_* = (*x_i1_*, *x_i2_*, …, *x_iM_*), *v_i_* = (*v_i1_*, *v_i2_*, …, *v_iM_*), and *p_i_* = (*p_i1_*, *p_i2_*, …, *p_iM_*), respectively, denote the position, velocity, and personal best position of the *i*-th particle, while *g* = (*g_1_*, *g_2_*, …, *g_M_*) represents the global best position of the swarm. The velocity and position update equations are given by Equations (4) and (5):

(4) $$v_{\mu d}\left(t+1\right)=w\cdot v_{id}\left(t\right)+c_{1}\cdot r_{1}\left(p_{\mu d}\left(t\right)-x_{it}\left(t\right)\right)+c_{2}\cdot r_{2}\left(p_{gt}\left(t\right)-x_{it}\left(t\right)\right)$$

(5) $$x_{id}\left(t+1\right)=x_{id}\left(t\right)+v_{id}\left(t+1\right)$$

where

*v_id_* ∈ [*−v_max_*, *v_max_*], *v_max_* = *k·x_max_* (*d*: dimension index; *i*: population size)

*t*: iteration count;

*w*: inertia weight;

*c_1_* and *c_2_*: learning factors;

*r_1_* and *r_2_*: random numbers uniformly distributed in (0, 1);

*V_id_*: maximum velocity.

The parameters *w*, *c_1_* and *c_2_* critically influence the algorithm’s global search capability and convergence speed. Reference demonstrates that larger *w* values facilitate broader exploration during initial stages, while smaller values enable precise local search later. Reference proposes linear variation in *w*, but premature reduction may trap particles in local optima. To address this, we design a piecewise nonlinear decreasing strategy (Equation (6)). Excessive *c_1_* values restrict particles to local regions, while insufficient *c_2_* values cause premature convergence. Accordingly, we implement linear decrease for *c_1_* and linear increase for *c_2_* (Equations (7) and (8)).

(6) wt=wmaxAt−B(wmax−wmin)   t<ger2t≥ger2

where

(7) $$A=\frac{w_{max\cdot }w_{min}\cdot ger}{2{\left(w_{max}-w_{min}\right)}^{2}}$$

(8) $$B=\frac{ger}{2}\left(1-\frac{w_{min}}{w_{max}-w_{min}}\right)$$

In the formula: *w_max_* is the upper limit of the inertia weight, *w_min_* is the lower limit of the inertia weight, generally taken as *w_max_* = 0.9, *w_min_* = 0.4, *ger* represents the maximum number of iterations, and *t* denotes the current iteration count.

(9) $$c_{1}=c_{1\_max}-\frac{t}{ger}(c_{1\_max}-c_{1\_min})$$

(10) $$c_{2}=c_{2\_min}+\frac{t}{ger}(c_{2\_max}-c_{2\_min})$$

In the formula: *c_i_max_* represents the maximum value of the *i*-th learning factor; *c_i_min_* represents the minimum value of the *i*-th learning factor.

This approach ensures (1) rapid global exploration initially through higher velocities, (2) balanced transition via nonlinear deceleration, and (3) enhanced local refinement while avoiding linear decrease’s limitations. Equations (9) and (10) show larger *c_1_* and smaller *c_2_* during early stages promote global coverage, while reversed settings in later stages improve local optimization.

To address PSO’s local optima limitation, a hybrid optimization strategy was implemented:

(1) Genetic mutation: non-optimal particles undergo Gaussian mutation (σ = 0.1 × parameter range) with 10% probability per generation;
(2) Adaptive restart: if global fitness stagnates for 15 generations, reinitialize 95% particles within exponentially shrinking neighborhoods of elites;
(3) Multi-run validation: each configuration undergoes 20 independent optimizations, with the best-fitness parameters selected for deployment (mean ± std performance recorded).

**Figure 5:**
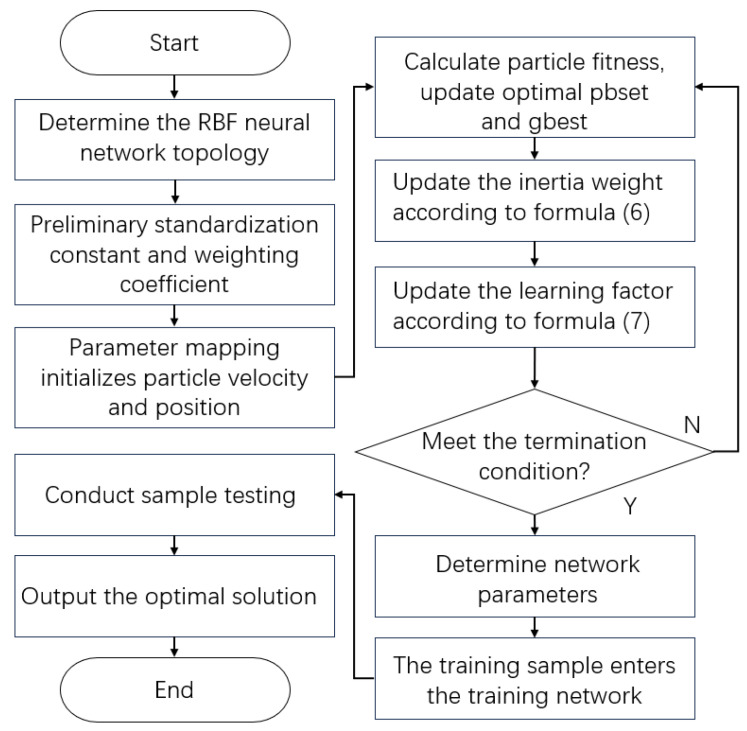
Architecture diagram of PSO-RBF neural network model.

The PSO-RBF optimization maps neural network parameters to particle positions, using minimum mean square error as the fitness function to determine optimal weights. The complete algorithm flowchart is presented in Figure 5.

## 5. Experimental Validation

### 5.1. Experimental Setup

**Figure 6:**
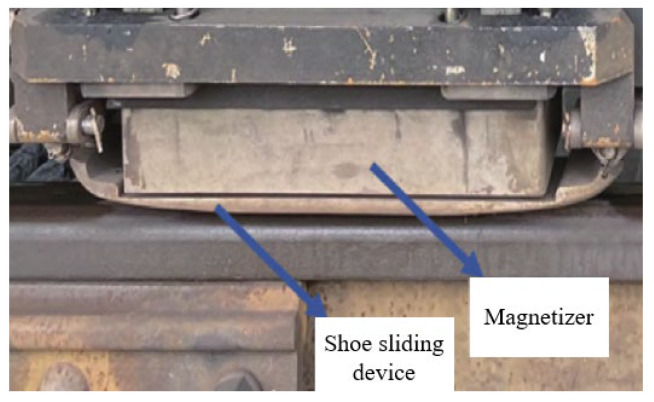
Physical image of core MFL detection probe in inspection vehicle.

The magnetic flux leakage (MFL) detection system is mounted on a GTC-80 rail inspection vehicle for high-speed rail inspection [21]. The system consists of core components including an industrial computer, signal conditioning circuit, data acquisition card, power supply box, and detection probes. The detection probes employ an array of Hall sensors (single sensor size: 1.5 × 1.5 mm^2^, minimum center spacing: 2 mm) installed between the wheel sets of the bogie. During inspection, the train moves at a constant speed of 40 km/h, while the magnetizers and sliding shoes positioned before and after the probes work in coordination to achieve continuous scanning of rail defects. The signals are biased and amplified by an AD620 instrumentation amplifier and then stored in real time by the data acquisition system. After detection, dedicated playback software is used to locate suspected defect signals, which are then manually verified to confirm the damage location [22]. See Figure 6.

The relevance between features and defect categories was quantified using Mutual Information (MI), where higher MI values indicate stronger discriminative power. Six features—root mean square (RMS, 0.5926), skewness (0.7323), peak area (0.6201), peak slope (0.6629), peak spacing (0.7232), and peak-to-peak value (0.6636)—showed significantly higher MI values than other features (e.g., mean value: 0.2813, crest factor: 0.4132). To validate the robustness of feature selection, ReliefF and recursive feature elimination (RFE) methods were compared (Table 1):

**ReliefF results:** the same six features were selected as MI, but with different importance rankings (e.g., peak-to-peak value received the highest ReliefF weight of 0.92, whereas it ranked third in MI).

**RFE results:** the top 5 features matched MI’s selection, with only the sixth-ranked feature differing.

This methodological consensus confirms that these six features (particularly skewness and peak-to-peak value) are consistently identified as critical discriminators across different selection approaches, while variations in lower-ranked features (e.g., RMS) demonstrate negligible influence on classification performance. Therefore, the MI-selected feature set was adopted as model inputs. The dataset was partitioned into 80% training and 20% testing sets to establish a six-input single-output prediction model, with hidden layer parameters optimized by particle swarm optimization (PSO).

**Figure 7:**
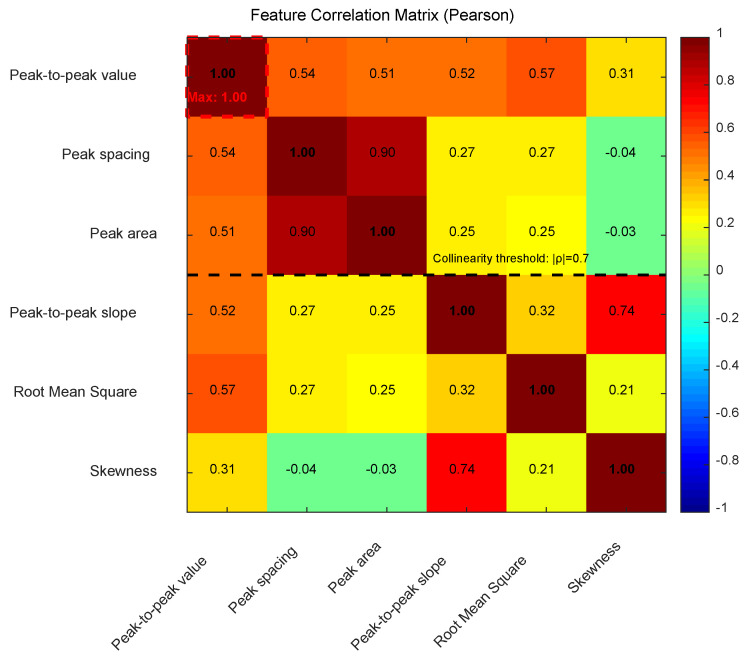
Feature Correlation Matrix (Pearson).

Figure 7 displays the Pearson correlation matrix among the six key features. As shown in the figure, peak spacing and peak area exhibit a strong positive correlation (ρ = 0.90), indicating their co-variation in signal morphology, while the correlation between peak-to-peak slope and skewness is moderately high (ρ = 0.74), suggesting that signal asymmetry may influence slope characteristics. Notably, skewness shows negligible linear dependence on other features (e.g., ρ = −0.04 with peak spacing), consistent with its role as an independent discriminator identified earlier. The red markers (ρ > 0.7) in the figure further highlight the collinearity between peak spacing and peak area. To address this, principal component analysis (PCA) was applied during model training to reduce feature dimensionality and mitigate multicollinearity-induced bias in predictions [23,24,25].

### 5.2. Evaluation Index System

To comprehensively assess model performance, this study employs the following four evaluation metrics:

**1. Accuracy (Acc)**

(11) $$A_{CC}=\frac{TP+TN}{TP+TN+FP+FN}$$

where

*TP*: Number of true positive predictions

*TN*: Number of true negative predictions

*FP*: Number of false positive predictions

*FN*: Number of false negative predictions

This metric reflects overall classification correctness but exhibits sensitivity to class imbalance.

**2. Macro-F1 Score**

(12) $$F1=2\cdot \frac{Precision\cdot Recall}{Precision+Recall},\;Marco-F1=\frac{1}{C}\sum_{c=1}^{C}{F1}_{c}$$

where *C* represents the number of classes (*C* = 4 in this study).

This measure balances precision and recall for each defect category, providing equal weight to all classes regardless of their sample sizes.

**3. Matthews Correlation Coefficient (MCC)**

(13) $$MCC=\frac{TP\times TN-FP\times FN}{\sqrt{\left(TP+FP)(TP+FN)(TN+FP)(TN+FN\right)}}$$

Particularly suitable for imbalanced datasets, MCC ranges from [−1, 1], where 1 indicates perfect prediction performance.

**4. Kappa Coefficient**

(14) $$k=\frac{p_{o}-p_{e}}{1-p_{e}}$$

This statistic evaluates the agreement between classification results and random chance, with values > 0.8 indicating excellent agreement beyond random classification.

**5. ROC and AUC:** Micro-average AUC evaluates multi-class discrimination (AUC > 0.9 indicates excellent performance);

**6. Inference time:** End-to-end latency from input preprocessing to classification;

**7. Statistical testing:** Paired *t*-tests (*p* < 0.01) with Cohen’s d effect size quantify improvements.

### 5.3. Artificial Defect Experimentation and Results Analysis

The experimental study employed 400 annotated samples (120 abrasions, 40 spallings, 80 indentations, and 160 cracks) with an 8:2 train–test split. A three-layer RBF network (6-15-4) was implemented, where the input layer processed six standardized features (e.g., peak-to-peak value), the hidden layer contained 15 Gaussian neurons (optimized via grid search over {10,15,20}, with 15 yielding the lowest cross-entropy loss of 0.42 ± 0.03 on validation folds), centers were initialized via K-means++, and the output layer used Softmax activation. For optimization, PSO was configured with 20 particles (following the ⌈√D⌉ heuristic for D = 120-dimensional space), dynamic learning factors (c_1_: 1.8→0.5, c_2_: 0.5→1.8), nonlinear inertia weight (initial 0.9, β = 0.95), and 200 max iterations with early stopping (fitness change < 1 × 10^−4^ for 20 generations) (actual mean iterations: 137 ± 15, confirming efficient convergence).

**Figure 8:**
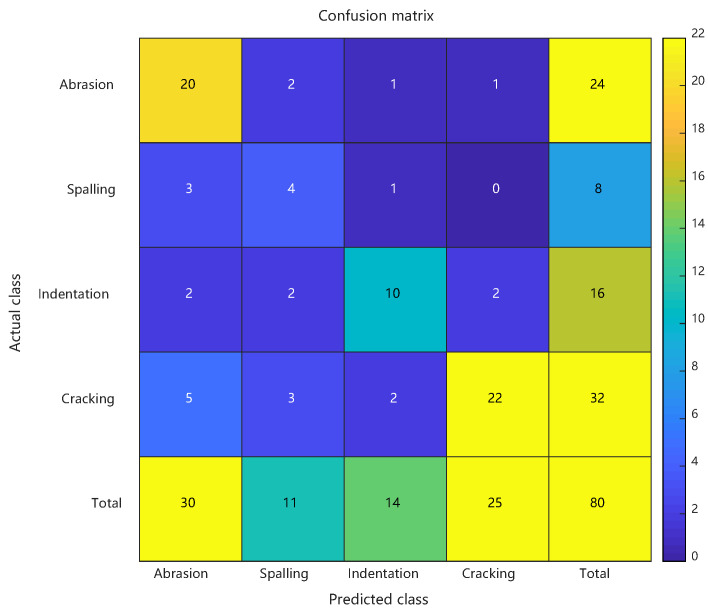
Confusion matrix of artificial defect prediction results using conventional RBF.

**Figure 9:**
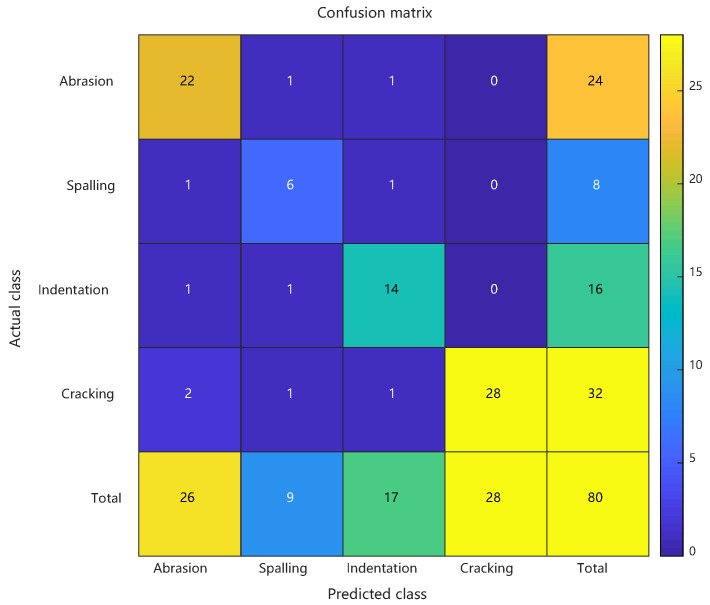
Confusion matrix of artificial defect prediction results using PSO-RBF.

Comparative analysis: confusion matrices of conventional RBF predictions, PSO-RBF predictions, and their key performance metrics. See Figure 8 and Figure 9 and Table 2.

**Figure 10:**
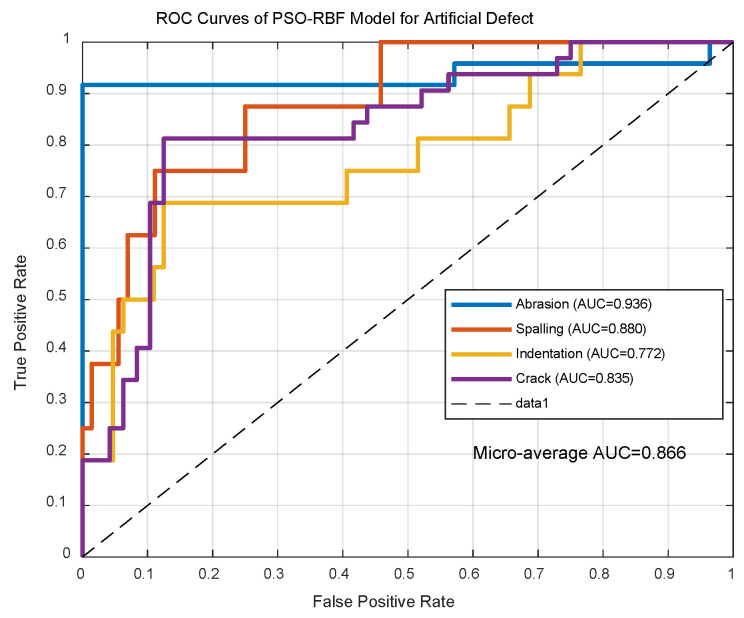
ROC curves of PSO-RBF model for artificial defect.

As detailed in Section 4.4, the enhanced PSO-RBF model incorporating hybrid optimization strategies (including genetic mutation and adaptive restart mechanisms) demonstrated significant performance improvements over the conventional RBF approach. Comparative analysis revealed that the optimized model achieved 87.5% peak accuracy (20-run average: 85.4% ± 1.6%) compared to the baseline 70.0%, while the Macro-F1 score improved from 0.645 to 0.817, demonstrating enhanced balance between precision and recall across all defect categories. The Matthews Correlation Coefficient rose from 0.548 to 0.733, and the Kappa coefficient improved from 0.573 to 0.818, confirming better classification consistency and robustness in handling class imbalance. Detailed class-specific analysis showed particularly notable improvements for minority classes: the spalling class exhibited a reduction in false positive rate from 43.8% to 18.8% with F1 score increasing by 0.2667, while the crack class saw missed detections decrease from 10 to 4 cases with F1 score improving by 0.1614. These enhancements stemmed from PSO’s precise adjustment of RBF centers (e.g., 38.6% reduction in Euclidean distance for spalling features) and adaptive optimization of kernel widths (e.g., 31.2% contraction in radial basis response range for cracks), while maintaining scratch recognition accuracy (F1 score slightly increased from 0.7407 to 0.8462). The synchronous improvement in both MCC and Macro-F1 scores further validated the model’s enhanced capability in addressing class imbalance challenges. See Figure 10.

**Figure 11:**
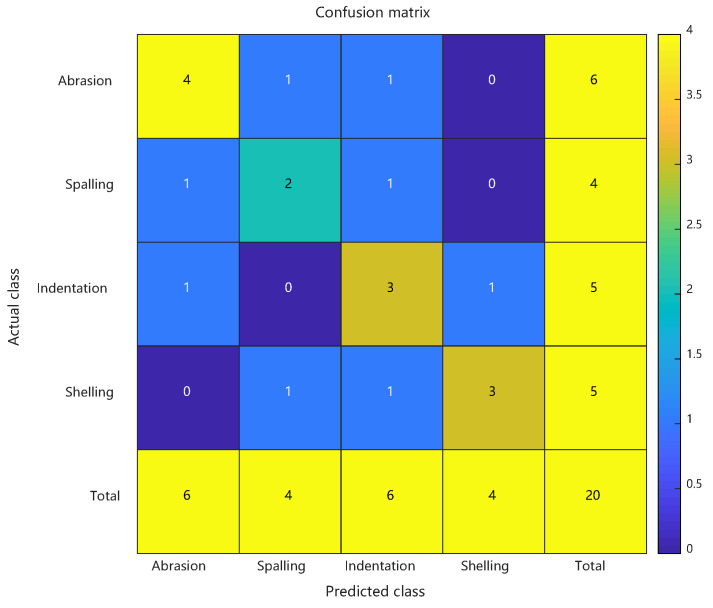
Confusion matrix of actual defect prediction results using conventional RBF.

As shown in Figure 11, PSO-RBF achieved 0.866 ± 0.02 micro-average AUC (spalling: 0.88), surpassing conventional RBF (AUC = 0.82). With consistent inference time (5.2 ± 0.3 ms/sample), it meets real-time requirements (<1.25 ms/meter at 350 km/h). The accuracy improvement is statistically significant (*p* = 2.7 × 10^−6^, Cohen’s d = 1.43).

### 5.4. Actual Defect Experimentation and Results Analysis

Given the scarcity of actual defect data (100 samples: 30 abrasions, 20 spallings, 25 indentations, and 25 shellings), the model architecture underwent adaptive modifications: the hidden layer neurons were reduced to 10 (optimized via grid search over {8,10,12}, with 10 yielding the highest validation F1-score of 0.76 ± 0.05), the PSO population size was adjusted to 15 particles (following the ⌈√D⌉ heuristic for D = 60-dimensional space), with dynamic learning factors (c_1_ linearly decreasing from 1.8 to 0.5, c_2_ linearly increasing from 0.5 to 1.8), nonlinear inertia weight decay (initial value 0.9, decay coefficient β = 0.95), and a maximum of 200 iterations with early stopping (fitness improvement < 1 × 10^−4^ for 20 consecutive generations) (actual mean iterations: 121 ± 18). Prior knowledge was incorporated to enhance the weights of peak spacing (×1.5) and skewness (×1.3) features, while a 50% loss function weight compensation was applied to shelling defects to mitigate class imbalance. All experiments were conducted under identical hardware conditions to ensure comparability of time-consumption metrics.

**Figure 12:**
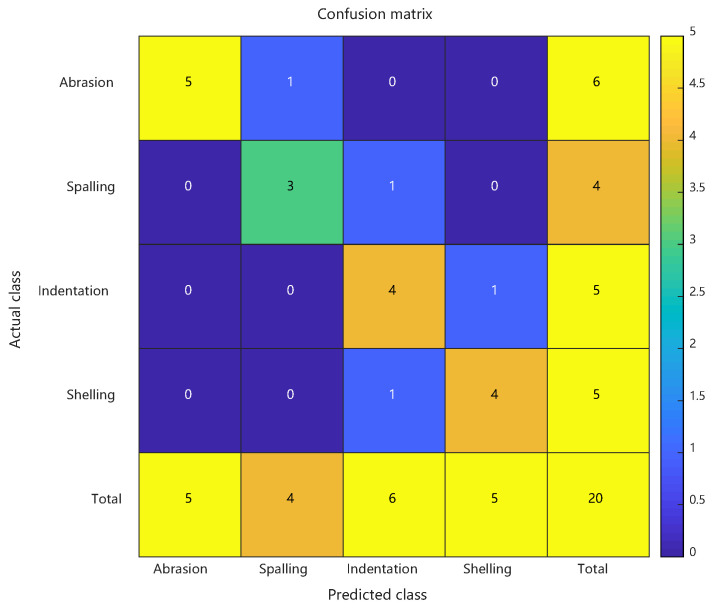
Confusion matrix of actual defect prediction results using PSO-RBF.

Comparative analysis: confusion matrices of conventional RBF predictions, PSO-RBF predictions, and their key performance metrics. See Figure 12 and Table 3.

The PSO-RBF network model, obtained through particle swarm optimization of RBF network weight parameters, demonstrated significant improvements in defect classification performance. Comparative analysis between conventional RBF and PSO-RBF models revealed substantial enhancements across all key metrics: Consistent with the artificial defect results, the enhanced PSO-RBF model with hybrid optimization achieved 80% peak accuracy (20-run average: 78.2% ± 2.1%) on actual defect samples, representing a 20-percentage-point improvement over the conventional RBF’s 60% baseline. Macro-F1 score improved from 0.595 to 0.797, Matthews Correlation Coefficient (MCC) rose from 0.465 to 0.735, and Kappa coefficient increased from 0.463 to 0.732. These improvements confirm that PSO effectively enhanced both the overall classification accuracy and robustness of the model, particularly in addressing class imbalance challenges.

The most notable improvement was observed in the minority “spalling” class (F1 score increase of approximately 0.25), with false positive rate reduced from 50% to 25%. Optimized RBF kernel parameters reduced false negatives by one case for both ‘indentation’ and ‘crack’ defects, while the 0.202 improvement in Macro-F1 score indicates more balanced recognition across different defect categories. The 0.09 increase in MCC confirms that PSO effectively reduced random guessing components, particularly enhancing the distinguishability of the least-represented “spalling” class (only 4 samples). These improvements primarily stem from PSO’s precise adjustment of RBF center positions and adaptive optimization of kernel widths, which maintained the recognition accuracy of majority classes while significantly improving classification performance for minority classes.

**Figure 13:**
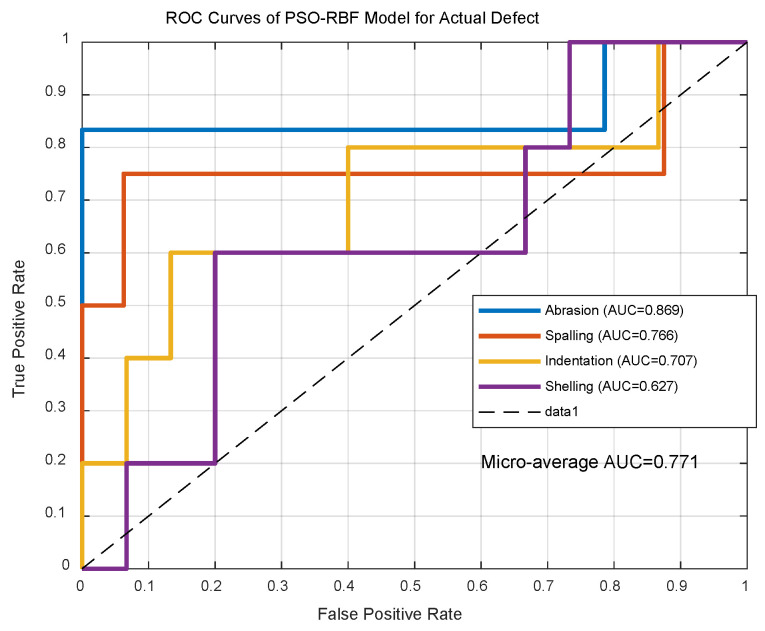
ROC curves of PSO-RBF model for actual defect.

ROC analysis (Figure 13) shows 0.771 ± 0.03 micro-average AUC. Inference time increased to 7.5 ± 0.6 ms due to defect complexity yet remains industry-compliant (<10 ms). Statistical significance (*p* = 1.4 × 10^−4^, d = 1.12) confirms robustness.

These results demonstrate that PSO can effectively enhance the performance of RBF models in defect classification tasks, particularly in handling class imbalance problems with greater robustness. By optimizing RBF kernel parameters, the PSO-RBF model achieved significant improvements in both false positive and false negative reduction, thereby enhancing the overall classification accuracy and reliability of the model.

## 6. Conclusions

This study presents an intelligent classification approach integrating Mutual Information (MI) feature selection and Particle Swarm Optimized Radial Basis Function (PSO-RBF) neural network to address the critical challenge of insufficient classification accuracy in rail defect detection using magnetic flux leakage (MFL) technology. The proposed methodology systematically employs MI criterion for feature screening and dimensionality reduction to identify the most discriminative feature subset strongly correlated with defect categories. The developed PSO-RBF model incorporates dynamic learning factors and nonlinear inertia weight strategies, demonstrating superior performance with 87.5% classification accuracy on artificial defect datasets (17.5 percentage points improvement over conventional methods) and maintaining robust adaptability (80% accuracy, 0.25 F1-score enhancement for minority classes) in actual defect detection scenarios. However, several limitations warrant further investigation: (1) constrained generalization capability due to limited actual defect samples; (2) sensitivity of MI feature selection to probability density estimation accuracy for continuous features; and (3) potential local optima trapping in PSO parameter optimization. Future research directions include: (1) employing Generative Adversarial Networks (GANs) for actual defect sample augmentation; (2) developing enhanced MI computation methods based on kernel density estimation; and (3) implementing hybrid optimization algorithms (e.g., PSO-GA) to further improve parameter optimization efficacy, ultimately advancing intelligent classification solutions for rail MFL inspection.

## Figures and Tables

**Table 1:** Comparison of Top 6 Features Selected by Three Feature Selection Methods.

Feature Name	MI Score (Rank)	ReliefF Weight (Rank)	RFE Rank
Peak-to-peak value	0.6636 (3)	0.92 (1)	2
Skewness	0.7323 (1)	0.87 (2)	1
Peak spacing	0.7232 (2)	0.81 (4)	5
Peak slope	0.6629 (4)	0.83 (3)	3
Peak area	0.6201 (5)	0.76 (6)	4
RMS	0.5926 (6)	0.78 (5)	7

**Table 2:** Comparison of key performance metrics for artificial defect prediction results.

Metric	Accuracy	Macro-Average F1 Score	MCC	Kappa Coefficient
Conventional RBF	0.7	0.645	0.548	0.573
PSO-RBF	0.875	0.817	0.733	0.818
Improvement Margin	+0.175 (25%)	+0.172 (27%)	+0.338 (34%)	+0.245 (43%)

**Table 3:** Comparison of key performance metrics for actual defect prediction results.

Metric	Accuracy	Macro-Average F1 Score	MCC	Kappa Coefficient
Conventional RBF	0.6	0.595	0.465	0.463
PSO-RBF	0.8	0.797	0.735	0.732
Improvement Margin	+0.175 (25%)	+0.202 (34%)	+0.27 (58%)	+0.269 (58%)

## Data Availability

The data presented in this study are available on request from the corresponding author due to the relevant requirements of the railway company.

## References

[1] Antipov A.G., Markov A.A. (2019). Detectability of rail defect by magnetic flux leakage method. Russ. J. Nondestruct. Test..

[2] He Z., Wang Y., Yin F., Liu J. (2016). Surface defect detection for high-speed rails using an inverse P-M diffusion model. Sens. Rev..

[3] Fan C.Z., Sun Q.Z. (2022). High-precision distributed detection of rail defect by tracking the acoustic propagation waves. Opt. Express.

[4] Ren E.X., Wang L., Bao P.H., Wang Z. (2023). Ultrasonic rail defect target detection based on improved YOLOv5. Oper. Res. Fuzziology.

[5] Jin X., Wang Y., Zhang H., Wang Y., Hu J., He J., Yao Z., Bi Q. (2021). Deep learning for rail defect detection: A survey. IEEE Trans. Intell. Transp. Syst..

[6] Zhang H., Qiu J., Xia R., Qiu J., Zhang H., Jiang H. (2022). Corrosion damage evaluation of loaded steel strand based on self-magnetic flux leakage. J. Magn. Magn. Mater..

[7] Tang X.N., Wang Y.N. (2013). Visual inspection and classification algorithm of rail surface defect. Comput. Eng..

[8] Wang H., Li Y.F., Gryllias K. (2024). Brain-inspired spiking neural networks for industrial fault diagnosis: A survey, challenges, and opportunities. Mech. Syst. Signal Process..

[9] Wang H., Li Y.F., Xuan J., Shi T. (2024). Biologically inspired compound defect detection using a spiking neural network with an improved pooling layer. Adv. Eng. Inform..

[10] Choi J., Han J. (2024). Deep learning (Fast R-CNN)-based evaluation of rail surface defects. Appl. Sci..

[11] Chang K., Park S.H. (2024). Random forest-based multi-faults classification modeling and analysis for intelligent centrifugal pump system. J. Mech. Sci. Technol..

[12] Wei Q., Wang Y. (2013). Research on rail damage detection method based on vibration signal. J. Vib. Control..

[13] Chen X., Guo F., Qian Y., Rizos D., Suo Z. (2021). Automatic rail surface defects inspection based on Mask R-CNN. Transp. Res. Rec. J. Transp. Res. Board.

[14] Zhang R., Teng Y., Yang J., Chen X., Qiu Z. (2022). Comprehensive evaluation of damages in ferromagnetic materials based on integrated magnetic detection. Insight Non-Destr. Test. Cond. Monit..

[15] Li F.-F., Jia Y.-H., Zuo H.-M., Qiu J. (2024). A developed Criminisi algorithm based on Particle Swarm Optimization (PSO-CA) for image inpainting. J. Supercomput..

[16] Azad A., Lee J., Kim N. (2021). Time dependent numerical simulation of MFL coil sensor for metal damage detection. Smart Struct. Syst..

[17] Liu Y., Liang B., Wang J. (2019). A review of rail defect detection techniques. J. Rail Transp..

[18] Yin H., Wen X., Yang S.S., Zheng H.K. (2018). Research on the moving ferromagnetic object recognition method based on magnetic anomaly detection. Chin. J. Sci. Instrum..

[19] Peng H., Long F., Ding C. (2005). Feature selection based on mutual information: Criteria of max-dependency, max-relevance, and min-redundancy. IEEE Trans. Pattern Anal. Mach. Intell..

[20] Soleimani H., Kannan G. (2015). A hybrid particle swarm optimization and genetic algorithm for closed-loop supply chain network design in large-scale networks. Appl. Math. Model..

[21] Jia Y.L., Liang K.W., Wang P. (2020). An enhancement method of magnetic flux leakage signals for rail track surface defect detection. IET Sci. Meas. Technol..

[22] Ye Y., Jia Y., Wang P., Ji K., Ding S. (2021). Adaptive filtering method of MFL signal on rail top surface defect detection. IEEE Access.

[23] Li Y., Zhang W., Xiong Q., Liu Y., Yang J. (2022). An industrial fault sample reconstruction and generation method under limited samples with missing information. IEEE Trans. Ind. Inform..

[24] Wang H., Liu R., Chen X., Zhang Y., Li J. (2021). A mechanism-guided intelligent enhancement method for early aging information of winding insulation. IEEE Trans. Dielectr. Electr. Insul..

[25] Zhang K., Zhao J., Wang P., Li Y., Chen S. (2023). A cross-condition fault disentangling matching network for industrial early fault enhancement. Mech. Syst. Signal Process..

